# In memoriam: Professor Dan J. Stein (1962–2025): a global clinician, philosopher and bridge-builder

**DOI:** 10.1017/neu.2025.10049

**Published:** 2025-12-22

**Authors:** Gregers Wegener, Sophie Erhardt, Ole A. Andreassen, Bonginkosi Chiliza, Lukoye Atwoli, Akena Dickens

**Affiliations:** 1 Translational Neuropsychiatry Unit (TNU), Department of Clinical Medicine, Aarhus University, Aarhus N, 8200, Denmark; 2 Department of Affective Disorders, Aarhus University Hospital - Psychiatry, Aarhus N, 8200, Denmark; 3 Department of Physiology and Pharmacology, Karolinska Institutet, Stockholm, 17177, Sweden; 4 Center for Precision Psychiatry, Division of Mental Health and Addiction, Oslo University Hospital, Oslo, 0424, Norway; 5 Institute of Clinical Medicine, University of Oslo, Oslo, 0424, Norway; 6 Department of Psychiatry, School of Medicine, College of Health Sciences, University of KwaZulu-Natal, Durban, 4001, South Africa; 7 Brain and Mind Institute, the Aga Khan University, Nairobi, 00100, Kenya; 8 Department of Medicine, Medical College East Africa, the Aga Khan University, Nairobi, 00100, Kenya; 9 Department of Psychiatry, Makerere University College of Health Sciences, Kampala, 30101, Uganda

**Keywords:** Obituary, Dan J Stein, South Africa, mental Health, Africa

**Figure:**
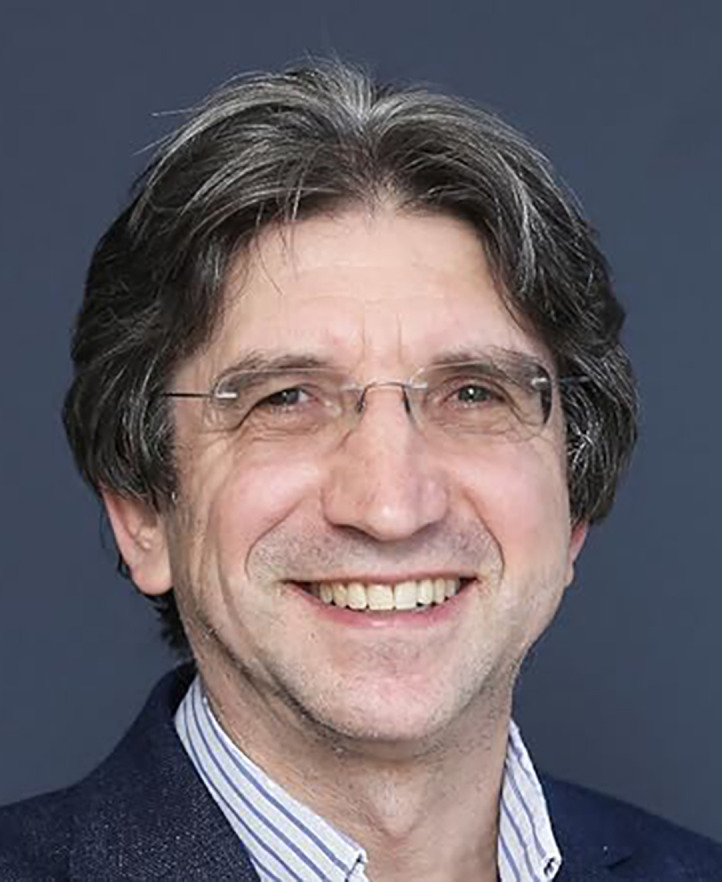
17 September 1962 – 6 December 2025

It is with profound sadness that we mark the passing of Professor *Dan J. Stein*, clinician, scientist, philosopher and a steadfast friend to the Scandinavian psychiatric community. While recognised globally as a leader in neuropsychopharmacology, *Dan* held a unique and cherished position as the architect of a vital bridge between African and Nordic psychiatry.

## A life of ethical science

Born in South Africa in 1962, *Dan* completed his medical training at the University of Cape Town before moving to the United States to avoid service in the apartheid-era military. He completed his residency and a post-doctoral fellowship at Columbia University, establishing himself as a rising star in anxiety and obsessive–compulsive disorders research.

Although his career took him abroad, his heart remained in South Africa. After the democratic election of Nelson Mandela, *Dan* returned home, a decision he described as an ethical commitment to help rebuild his country through science. Back in South Africa, he completed a PhD in clinical neuroscience, later followed by a second PhD in philosophy. He then established what became the South African Medical Research Council Unit on Anxiety and Stress Disorders at Stellenbosch University, which he directed from 1994 to 2005. This unit, the first dedicated mental health research unit of its kind in South Africa, carried out foundational work in anxiety neuroscience, launched some of the country’s first brain MRI and neurogenetics studies in psychiatry and led the South African Stress and Health (SASH) study, the first nationally representative survey of mental disorders on the African continent.

## The college pillars: AfCNP, SCNP and CINP

While his contributions and influence on the DSM-5 and ICD-11 are very well known and documented, *Dan*’s legacy within our community is defined by his visionary leadership in connecting the Global South with the Global North. *Dan* was the Founding President of the *African College of Neuropsychopharmacology (AfCNP).* He envisioned an organisation that would give African neuropsychopharmacology a home and a voice. Crucially, he did not view this mission in isolation. He worked to forge bonds between the AfCNP and the *Scandinavian College of Neuropsychopharmacology (SCNP)*, the *European College of Neuropsychopharmacology (ECNP),* and the *International College of Neuropsychopharmacology (CINP)*.

He facilitated a dynamic exchange where ideas, data and people moved between Africa, Scandinavia and the world. He ensured that *AfCNP* congresses became meeting places open for Nordic and Global colleagues to engage with African scholarship, fostering a spirit of true collaboration. The meetings became a home for African neuropsychopharmacology, helping to ensure that African perspectives and data shaped the global field.

This commitment to cross-continental dialogue was very visible in his dedication to *Acta Neuropsychiatrica*. As a valued member of the Editorial Board, *Dan* was not merely a name on the paper, he was an active facilitator who enriched the journal with his rigour and perspective. He championed *Acta Neuropsychiatrica* as a platform for African research, notably facilitating the publication of *AfCNP* congress abstracts within the journal, and a whole special issue with scientific papers from Africa. By doing so, he ensured that the work of emerging African scientists was visible to the Scandinavian and international readership, outlining the position of *Acta Neuropsychiatrica* as a vehicle for global scientific integration. His editorial wisdom helped position the journal at the intersection of biological research and clinical applicability.

*Dan*’s connection to the Nordic region extended beyond societies and journals into deep personal and institutional ties. He served as an Honorary Professor at *Aarhus University* in Denmark. In Aarhus, *Dan* was more than a visiting dignitary, he was a collaborator who worked to ‘build bridges between Denmark and South Africa’. He contributed to teaching and research, and his presence was always a highlight, showcasing his ability to merge complex neuroscience with humanistic philosophy.

## Leadership in African psychiatry and global mental health

Dan’s vision was never confined to one department or one country. He mentored generations of African clinicians and scientists whose work now spans addiction, child and adolescent psychiatry, liaison psychiatry, neurogenetics, neuroimaging, public mental health, psychopharmacology and psychotherapy – many of them now leaders in their own field.

He was a key architect of large collaborative projects that anchored African mental health in the global scientific conversation. The SASH study provided the first national prevalence data on common mental disorders in South Africa. The NeuroGAP Psychosis collaboration brought together investigators in Ethiopia, Kenya, South Africa and Uganda to address the profound underrepresentation of African genomes in psychiatric genetics, focusing on schizophrenia and bipolar disorder. He was also involved in global collaborations such as the World Mental Health Surveys, the ENIGMA neuroimaging consortium and the Psychiatric Genetics Consortium.

## A global giant

*Dan*’s scholarly output was astonishing in its breadth and depth. He authored or edited more than fifty books and more than two thousand journal articles and chapters, ranging from basic neuroscience to clinical trials, epidemiology, public mental health and philosophy of psychiatry. Bibliometric analyses consistently place him among the most influential psychiatrists worldwide.

He was recognised with major international and national honours, including the *Max Hamilton Memorial Award (*CINP*)*, the *SAMRC Platinum Award*, the *Lifetime Achievement Award* of the World Federation of Societies of Biological Psychiatry, the *John F.W. Herschel Medal* of the Royal Society of South Africa and election as a Fellow of the Royal College of Physicians and Surgeons of Canada.

## Integrating bench, bed and bundu

A defining feature of *Dan*’s career was his insistence that psychiatry is integrative. He moved fluently between bench (laboratory neuroscience), bed (clinical trials and treatment studies) and bundu (epidemiology, public mental health and policy in real-world African settings).

His work on anxiety, obsessive–compulsive and related disorders and trauma- and stressor-related disorders was both biologically sophisticated and acutely attuned to social determinants and human rights. He showed that high-quality neuroscience and high-impact public mental health research are not luxuries reserved for wealthy countries, but necessities for societies emerging from colonialism, apartheid, violence and inequality.

## Colleague, mentor and friend

For all his extraordinary achievements, we who worked with *Dan* will remember above all his quiet generosity, intellectual humility and unwavering collegiality. He answered emails from junior colleagues with the same care he gave to international committees; he read manuscripts with forensic attention but responded with warmth and encouragement. As co-workers in the editorial world, we were privileged to experience his combination of absolute rigour and unfailing kindness.

Professor *Dan J. Stein* leaves a void that cannot be filled. His work will continue in the departments he led, the units he founded, the colleges and journals he served, the students he taught and the lives of the patients whose suffering he helped to relieve. The bridges he built between the African and Scandinavian colleges are strong.

As we mourn his death, we pledge to honour his memory by keeping those bridges open, continuing the dialogue he started and maintaining the high standards he set for our journal and our field.

Our sincere thoughts are with his wife, Professor *Heather Zar*; children *Gabriella*, *Joshua* and *Sarah*; and grandson, *Rafa* – to whom we extend our deepest condolences and enduring respect.

- **Gregers Wegener,** Editor-in-Chief (*Acta Neuropsychiatrica*), Past-President (*SCNP*)
- **Sophie Erhardt,** President (*SCNP*)
- **Ole A. Andreassen**, Past-President (*SCNP*)
- **Bongikosi Chiliza,** President (*AfCNP*)
- **Lukoye Atwoli,** Past-President (*AfCNP*)
- **Akena Dickens,** Past-President (*AfCNP*)

## Abbreviations

- **AfCNP**: African College of Neuropsychopharmacology
- **CINP**: International College of Neuropsychopharmacology
- **ECNP**: European College of Neuropsychopharmacology
- **SCNP**: Scandinavian College of Neuropsychopharmacology

## Data Availability

Data availability is not applicable as no new data were created or analysed.

